# A Call for Consistency in the Official Naming of the Disease Caused by Severe Acute Respiratory Syndrome Coronavirus 2 in Non-English Languages

**DOI:** 10.1017/dmp.2020.169

**Published:** 2020-05-22

**Authors:** Lu Dong, Zhe Li, Isaac Chun Hai Fung

**Affiliations:** RAND Corporation, Santa Monica, CA, USA; Independent Researcher, Santa Monica, CA, USA; Department of Biostatistics, Epidemiology and Environmental Health Sciences, Jiann-Ping Hsu College of Public Health, Georgia Southern University, Statesboro, GA, USA

**Keywords:** COVID-19, infectious disease medicine, naming, SARS-CoV-2

## Abstract

We investigated the adoption of World Health Organization (WHO) naming of COVID-19 into the respective languages among the Group of Twenty (G20) countries, and the variation of COVID-19 naming in the Chinese language across different health authorities. On May 7, 2020, we identified the websites of the national health authorities of the G20 countries to identify naming of COVID-19 in their respective languages, and the websites of the health authorities in mainland China, Hong Kong, Macau, Taiwan and Singapore and identify their Chinese name for COVID-19. Among the G20 nations, Argentina, China, Italy, Japan, Mexico, Saudi Arabia and Turkey do not use the literal translation of COVID-19 in their official language(s) to refer to COVID-19, as they retain “novel” in the naming of this disease. China is the only G20 nation that names COVID-19 a pneumonia. Among Chinese-speaking jurisdictions, Hong Kong and Singapore governments follow the WHO’s recommendation and adopt the literal translation of COVID-19 in Chinese. In contrast, mainland China, Macau, and Taiwan refer to COVID-19 as a type of pneumonia in Chinese. We urge health authorities worldwide to adopt naming in their native languages that are consistent with WHO’s naming of COVID-19.

The disease caused by severe acute respiratory syndrome coronavirus 2 (SARS-CoV-2) was thought to be a type of pneumonia when it first emerged in Wuhan. Hence, the National Health Commission of China (NHC) announced on February 7, 2020, that the official name of the disease was *novel coronavirus pneumonia* (*NCP*; 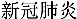; Xīnguàn fèiyán).^1^

On February 11, 2020, the World Health Organization (WHO) named the disease *coronavirus disease 2019* (COVID-19). The name does not include the word *pneumonia*. The WHO intentionally excluded the word *novel* to give “a standard format to use for any future coronavirus outbreaks.”^2^ The WHO provides official translation of COVID-19 into its 6 working languages (summarized in https://tinyurl.com/naming-table-s1); in Chinese, it is the literal translation of *COVID-19*: “2019 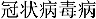.”

On February 21, 2020, the NHC announced that they were changing the English name of the disease to *COVID-19* but were keeping the Chinese name unchanged.^3^ Consistent with the name of *NCP*, the NHC provides detailed and rather stringent diagnostic criteria^4^ that may underdiagnose patients who are infected with SARS-CoV-2 but do not have explicit epidemiological links nor show any of the clinical symptoms (eg, fever and/or respiratory symptoms, imaging characteristics of NCP). For detailed diagnostic criteria, see https://tinyurl.com/naming-criteria-translation.

The Chinese official naming as *Xīnguàn fèiyán*/*NCP* is a semantic reflection of an outdated clinical understanding. As we now understand, COVID-19 cases may be asymptomatic or experience a broad spectrum of symptoms (eg, cardiovascular symptoms, neurological impairment), with pneumonia presenting only a portion of the spectrum.^5^ Given the evolving evidence, the NHC should reconsider their naming convention and case definition.

As a comparison, among the G20 nations, Argentina, China, Italy, Japan, Mexico, Saudi Arabia, and Turkey do not use the literal translation of *COVID-19* in their official language(s) to refer to this disease on their official websites, as they still retain “novel” in the naming. China is the only G20 nation that officially refers to COVID-19 as a type of pneumonia (Appendix). Among Chinese-speaking jurisdictions, Hong Kong and Singaporean Governments adopt the literal translation of *COVID-19* in Chinese. In contrast, mainland China, Macau, and Taiwan still refer to COVID-19 as a type of pneumonia in Chinese on their official websites (https://tinyurl.com/naming-table-s2).

The discrepancy between the Chinese and English naming of the illness, and the corresponding diagnostic criteria of COVID-19 used in China, should be acknowledged by the scientific community. It is especially important when we interpret results from Chinese studies concerning COVID-19, as the Chinese samples may have skewed heavily toward patients presented with pneumonia. Direct translation of *Xīnguàn fèiyán/NCP* into *COVID-19*, or viceversa, is problematic as the 2 entities are not equivalent.

Consistent naming will facilitate comparisons of COVID-19-related research findings across languages and countries. We call upon the WHO to ensure all member states to settle on the same naming of *COVID-19* in their respective languages. Likewise, we urge the NHC to properly adopt the literal translation of *COVID-19* into the Chinese language and to update the diagnostic criteria accordingly.

## Supplementary material

For supplementary material accompanying this paper visit http://dx.doi.org/10.1017/dmp.2020.169. click here to view supplementary material
