# Supplementary material for: A Call for Consistency in the Official Naming of the Disease Caused by Severe Acute Respiratory Syndrome Coronavirus 2 in Non-English Languages
Source: Disaster Med Public Health Prep. 2020 May 22:1–2. doi: 10.1017/dmp.2020.169 (PMC7322147; doi:10.1017/dmp.2020.169)
Supplement: Supplementary file 1 [file S193578932000169Xsup001.docx]

**Online Appendix**: **A Call for Consistency in Official Naming of the Disease Caused by SARS-CoV-2 in Non-English Languages**

Authors: Lu Dong, PhD; Zhe Li, MS; Isaac Chun-Hai Fung, PhD

**Table. G20 Nations Official Naming of the Disease Caused by SARS-CoV-2**

| Nations | Language ^a^ | Disease name in native language | Back translation to English | Source ^c^ |
| --- | --- | --- | --- | --- |
| Argentina | Spanish | Nuevo Coronavirus (COVID-19) | Novel Coronavirus (COVID-19) | [https://www.argentina.gob.ar/salud](https://www.argentina.gob.ar/salud/coronavirus-COVID-19) |
| Australia | English | Coronavirus (COVID-19) | Coronavirus (COVID-19) | <https://www.health.gov.au/> |
| Brazil | Portuguese | Coronavírus (COVID-19) | Coronavirus (COVID-19) | <https://coronavirus.saude.gov.br/> |
| Canada | English/  French | Coronavirus disease (COVID-19) /  Maladie à Coronavirus (COVID-19) | Coronavirus disease (COVID-19) | [https://www.canada.ca/en/public-health/](https://www.canada.ca/en/public-health/services/diseases/coronavirus-disease-covid-19.html) |
| China | Chinese | 新型冠状病毒肺炎^b^ | Novel Coronavirus Pneumonia | <http://www.nhc.gov.cn/> |
| France | French | Coronavirus (COVID-19) | Coronavirus (COVID-19) | <https://solidarites-sante.gouv.fr/> |
| Germany | German | Coronavirus SARS-CoV-2 | Coronavirus SARS-CoV-2 | <https://www.bfarm.de/> |
| India | Hindi | Coronavirus disease (COVID-19) | Coronavirus disease (COVID-19) | <https://www.mohfw.gov.in> |
| Indonesia | Malay | Coronavirus disease (COVID-19) | Coronavirus disease (COVID-19) | <https://www.kemkes.go.id/> |
| Italy | Italian | Nuovo coronavirus (COVID-19) | Novel Coronavirus (COVID-19) | <http://www.salute.gov.it/> |
| Japan | Japanese | 新型コロナウイルス感染症 | Novel Coronavirus Disease | <https://www.mhlw.go.jp/> |
| Mexico | Spanish | Nuevo Coronavirus (COVID-19) | Novel Coronavirus (COVID-19) | [https://coronavirus.gob.mx](https://coronavirus.gob.mx/) |
| Russia | Russian | коронавирусной инфекции (COVID-19) | Coronavirus Infection (COVID-19) | [http://government.ru/](http://government.ru/support_measures/) |
| Saudi Arabia | Arabic | فيروس كورونا الجديد ( كورونا COVID-19 ) | Novel Coronavirus (COVID-19) | [https://www.moh.gov.sa/](https://www.moh.gov.sa/Pages/default.aspx) |
| South Africa | English | Corona Virus (COVID-19) | Corona Virus (COVID-19) | <http://www.health.gov.za/> |
| South Korea | Korean | 코로나바이러스감염증-19 | Coronavirus Infection - 19 | http://www.mohw.go.kr/ |
| Turkey | Turkish | Yeni Koronavirüs | Novel Coronavirus | <https://www.saglik.gov.tr/> |
| UK | English | Coronavirus (COVID-19) | Coronavirus (COVID-19) | <https://www.nhs.uk/> |
| USA | English | Coronavirus Disease 2019 (COVID-19) | Coronavirus Disease 2019 (COVID-19) | <https://www.cdc.gov/> |

*Notes.* ^a^ Language is either the official language(s) or, in the case of multiple official languages, the language used in the official website. ^b^ The Chinese NHC published the announcement on February 21, 2020, which informed the national jointed mechanism for Novel Coronavirus Pneumonia containment that the Chinese NHC had made the decision to change the English naming of the Novel Coronavirus Pneumonia to COVID-19 to be consistent with the WHO. While the Chinese naming should remain unchanged.^7^ ^c^ Access date is May 7, 2020 for the websites listed under *sources*.
